# Evaluation of the antileishmanial potency, toxicity and phytochemical constituents of methanol bark extract of *Sterculia villosa*

**DOI:** 10.1080/13880209.2017.1285946

**Published:** 2017-02-07

**Authors:** Antu Das, Manash C. Das, Niranjan Das, Surajit Bhattacharjee

**Affiliations:** a Department of Molecular Biology & Bioinformatics, Tripura University (A Central University), Suryamaninagar, India;; b Department of Chemistry, Netaji Shubhas Mahavidyalaya, Udaipur, India

**Keywords:** Leishmanicidal, free radicals, lipid peroxidation, DNA fragmentation

## Abstract

**Context:** Visceral leishmaniasis is a protozoan disease caused by *Leishmania donovani* parasite. The genus *Sterculia* (Malvaceae) possesses ethnobotanical potential against this protozoan infection.

**Objective:** Determining the potential role of methanol bark extracts from *Sterculia villosa* Roxb (SVE) and its phytoconstituents against *Leishmania donovani* promastigotes.

**Materials and methods:** SVE was analysed by TLC, UV–Vis, IR spectroscopy and biochemical assays. Antileishmanial potential of SVE (0.5–130 μg/mL for 72 h) was characterized by MTT assay. Fluorescent microscopy was performed to validate the IC_50_ dose. To determine the effect of SVE on promastigotes, reactive oxygen species (ROS) and superoxide generation, lipid peroxidation and DNA fragmentation assays were performed. Molecular aggregation of compounds was determined by atomic force microscopy (AFM). Extent of cytotoxicity of SVE at IC_50_ dose was determined against RAW 264.7 macrophages, peritoneal macrophages and murine RBCs. *In vivo* cytotoxicity of SVE was evaluated in BALB/c mice.

**Result:** SVE exhibited reverse dose dependent antileishmanial activity when 130–0 μg/mL doses were tested against promastigotes. The IC_50_ and IC_70_ values were found to be 17.5 and 10 μg/mL, respectively. SVE at IC_50_ dose demonstrated elevated level of ROS, superoxide, lipid peroxidation and DNA fragmentation against promastigotes with no cytotoxicity. AFM analysis suggested increasing size of molecular aggregation (31.3 nm < 35.2 nm < 2.93 μm) with increase in concentration (10 μg < 17.5 μg < 130 μg).

**Discussion and conclusions:** The study elucidates the antileishmanial potential of SVE against *Leishmania donovani* promastigotes by exerting oxidative stress and DNA damage. In sum, SVE can be explored as an immunotherapeutic candidate against leishmaniasis and other infectious diseases.

## Introduction

Leishmaniasis, a parasitic disease caused by the protozoan *Leishmania donovani*, affects 14 million people directly among the ∼350 million people who live in actively *Leishmania* transmitted areas globally (Pace 2014). Moreover, 0.2–0.4 million people are affected by visceral leishmaniasis (VL) per year; of which, more than 90% are in India, Bangladesh, Sudan, Ethiopia and Brazil, with 10–20% mortality estimated in poor areas (Alvar et al. 2012). Visceral leishmaniasis or kalazar is the most severe form of leishmaniasis with many adverse symptoms including irregular fever, weight loss, anaemia, enlarged spleen and liver. The severity of this disease is further accelerated by the exposure of HIV-VL co-infection (Desjeux 1999; Chappuis et al. 2007). There are a few available drugs that are widely used for the treatment of VL, namely pentavalent antimonials, amphotericin B (AmpB) and lipid formulations of AmpB. All of these drugs have many limitations such as long course of treatment, toxic side effects, and high cost (Iwu et al. 1994). Moreover, chemo-resistance of *L. donovani* against these classical drugs has further worsened the treatment procedure (Sen et al. 2004; Roy et al. 2008). So, the need to develop new potent, cheaper and less toxic drugs for the treatment of VL is important.

There is growing interest in plant derived natural products, which are less toxic and safer as bioactive agents for which they are used widely all over the world (Iwu et al. 1994). India is rich in traditional medicinal plants with a vast biodiversity which provides a good opportunity to explore the flora to search new, potent, less toxic and safe therapeutic agents. *Sterculia villosa* Roxb (Malvaceae) is an ethnomedicinally significant plant. Traditionally, the whole plant possesses diuretic, cooling, aphrodisiac and anti-inflammatory properties (Kumar et al. 2004; Namsa et al. 2009). Sirup, prepared from the petiole of the plant, along with water and sugar, is given to treat urinary problems and rheumatism. Several parts of *S. villosa*, including the bark and petiole, are used as a remedy in seminal weakness. Root infusion is taken as food adjunct while the whole plant extract is useful for skin diseases (Kunwar et al. 2010). The plant also has anthelmintic (Haque et al. 2012), anti-inflammatory, antidiabetic (Hossain et al. 2012), antimicrobial, membrane stabilization and antithrombotic properties (Tania et al. 2013). The genus *Sterculia* also possesses antiprotozoal activity (Nwodo et al. 2015).

In the present study, the phytochemical constituents of methanol bark extract of *Sterculia villosa* (SVE) was evaluated. Following that, SVE was investigated for *in vitro* leishmanicidal potential against *Leishmania donovani* promastigotes. Further, the SVE was assayed for *in vitro* as well as *in vivo* toxicity.

## Materials and methods

### Chemicals

Foetal bovine serum (FBS) was purchased from Gibco, Bangalore, India; potassium bromide (KBr), AmpB, H_2_DCFDA were purchased from Sigma-Aldrich, St. Louis, MO; Triton X-100, sodium dodecyl sulphate (SDS) were purchased from Bio-Rad, Hercules, CA. All other chemicals were purchased from Himedia, Mumbai, India.

### Plant material

The stem bark of *Sterculia villosa* was collected from Suryamaninagar region, Tripura University, Tripura, India in August 2014. The plant was identified by Prof. B. K. Datta, Taxonomist, Department of Botany, Tripura University. A voucher specimen (Code No. TR0115) was deposited in the herbarium of Department of Botany, Tripura University and an accession no. 0495 was assigned to the specimen. *Sterculia villosa* bark was washed, cut into small pieces and then shade dried for 30 days.

### Preparation of plant extract

Air dried small pieces of *S. villosa* (3.3 kg) bark was extracted with MeOH (6.0 L each) at room temperature (three times) for seven days. The whole mixture was then filtered through a fresh cotton plug followed by a Whatman No. 1 filter paper. Following filtration, the volume of filtrate was reduced using rotary evaporator (Superfit Rotavat, model no. PBV-7D, Superfit Continental Pvt Ltd., Mumbai, India) and under reduced pressure at 45–50 °C and dried in vacuum oven at 45 °C. Likewise 113 g (3.42%) of crude methanol bark extract of *Sterculia villosa* (SVE) was prepared. SVE (5 mg/mL) was prepared in dimethyl sulphoxide (DMSO) as working stock for several biochemical colour tests.

### Thin layer chromatographic study

TLC is one of the most widely used and potent technique to separate individual component from mixture of phytochemicals. The TLC plates were prepared by silica gel G to observe the separation of individual phytochemicals as a single spot from the selected crude bark extract employing varying solvent polarities such as ethyl acetate in petroleum ether; chloroform in ethyl acetate; chloroform in methanol and water in methanol. The developed TLC plates were visualized with iodine staining to assess the presence of different types of phytochemicals/compounds in SVE (Rajkumar et al. 2012).

### Ultra violet (UV) and Fourier transform infrared (FTIR) spectrophotometric analysis

SVE was examined using UV–Vis and FTIR spectrophotometer for phytoconstituent analysis. For UV–Vis spectrophotometric analysis, the SVE powder was dissolved in DMSO at a concentration of 0.1 μg/mL. Continuous absorbances at wavelength ranging from 200 to 800 nm were measured using UV–visible spectrophotometer (Perkin Elmer, Waltham, MA). Further, FTIR spectroscopy was used to identify the characteristic functional groups in the SVE. A small quantity (5 mg) of the extract was dispersed in 100 mg dry KBr. The mixture was thoroughly mixed in a mortar and pressed at pressure of 6 bars within 2 min to form a thin disc of KBr. Then the disc was placed in a sample cup of a diffuse reflectance accessory. The IR spectrum was obtained using Perkin Elmer 2000 infrared spectrophotometer. The sample was scanned from 4000 to 400 cm^−1^ for 16 times to increase the signal to noise ratio using infrared spectrophotometer (Perkin Elmer 2000). Each analysis was repeated at least four times for the spectrum confirmation.

### Phytochemicals analyses

#### Test for flavonoids

*Alkaline reagent test*. The extract (1 mL) was taken in a test tube, and a few drops of dilute sodium hydroxide (NaOH) were added. Intense yellow colour appeared which became colourless on addition of few drops of dilute acid (HCl). This indicated the presence of flavonoids (De et al. 2010).

*Shinoda test*. The extract (1 mL) was treated with few fragments of magnesium ribbon and then concentrated hydrochloric acid was added drop wise. Appearance of red colour demonstrated the presence of flavonoids (Firadouse & Alam 2011).

#### Test for alkaloid

*Mayer’s reagent*. To the extract (1 mL), 2 mL of Mayer's reagent (mercuric chloride (2.72 g) dissolved in distilled water (120 mL) and separately potassium iodide (10 g) dissolved in distilled water (40 mL) were mixed together and volume made up to 200 mL with distilled water) was added. Appearance of a dull white precipitate revealed the presence of alkaloids (Firadouse & Alam 2011).

*Wagner’s reagent*. To the 1 mL of extract, 2 mL of Wagner's reagent (2 g of iodine and 6 g of potassium iodide dissolved in 100 mL of water) was added. The formation of a reddish brown precipitate indicated the presence of alkaloids (Firadouse & Alam 2011).

#### Test for tannin

*Ferric chloride (FeCl_3_) test*. The extract (0.5 mL) was taken in test tube and treated with few drops of diluted FeCl_3_. Appearance of blue or greenish-black colour that changed to olive green following addition of more FeCl_3_ indicated the presence of tannins (Yusuf et al. 2014).

*Gelatin test*. To the 1 mL of extract solution, 0.5 mL of 1% gelatin solution containing 10% sodium chloride was added. Formation of a white coloured precipitate confirmed the presence of tannins (De et al. 2010).

#### Test for sterol

*Liebermann–Burchard test*. To 1 mL of extract, 1 mL of glacial acetic acid, 1 mL of acetic anhydride and two drops of concentrated sulphuric acid (H_2_SO_4_) were added. Appearance of red, followed by blue and finally bluish green colour, indicated the presence of steroids (Firadouse & Alam 2011).

*Salkowski test*. The extract (1 mL) was treated with few drops of concentrated H_2_SO_4_ and 0.5 mL of chloroform. The solution was then shaken well and allowed to stand for some time. Appearance of red colour at the lower layer indicated the presence of steroids (De et al. 2010).

#### Test for anthraquinone

*Borntrager's test*. The extract (1 mL) was taken in a test tube and 1 mL of chloroform was added and shaken for 5 min. The mixture was filtered and the filtrate was then shaken with equal volume of 10% ammonia solution. A pink, red or violet colour in the aqueous layer after shaking indicated the presence of free anthraquinones (Yusuf et al. 2014).

*Test for phenol*. The extract (1 mL) was taken in a test tube and 2 mL of distilled water was added, followed by few drops of 10% FeCl_3_. Appearance of blue or green colour indicated the presence of phenols (Ganatra et al. 2012).

*Test for terpenoid*. In a test tube, 1 mL of extract was treated with 0.5 mL of chloroform and 0.5 mL of concentrated H_2_SO_4_. Formation of a reddish brown precipitate colouration at the interface indicated the presence of terpenoids (Ganatra et al. 2012).

*Test for anthocyanin*. To the 1 mL of extract, 0.5 mL of 10% NaOH was added. Formation of blue colour indicated the presence of anthocyanins (Firadouse & Alam 2011).

#### Test for reducing sugar

*Fehling’s solution test*. The extract (1 mL) was mixed with 1 mL of Fehling’s solution (Fehling's A: copper sulphate in distilled water and Fehling's B: potassium tartarate and NaOH in distilled water) and boiled for 5 min. Formation of brick red precipitate of cuprous oxide demonstrated the positive test for reducing sugar (De et al. 2010).

*Benedict’s test*. The extract (0.5 mL) was taken in a test tube and treated with 2 mL of Benedict’s solution. The solution was then boiled for 5 min. The appearance of brick red precipitate confirmed the presence of carbohydrates (Firadouse & Alam 2011).

*Test for quinone*. To 1 mL of extract, 1 mL of concentrated H_2_SO_4_ was added. Formation of red colour indicated the presence of quinones (Ganatra et al. 2012).

#### Test for saponin

*Froth formation test*. Extract solution (1 mL) was diluted with distilled water to 5 mL and shaken in a graduated cylinder for 15 min. Development of stable foam suggested the presence of saponins (Ganatra et al. 2012).

### Parasites

Promastigote forms of *L. donovani* (MHOM/IN/1983/AG83) were a kind gift from Dr. Bhaskar Saha, National Centre for Cell Science, Pune, India. Promastigote forms of the parasites were cultured *in vitro* at 22 °C in M199 liquid media (pH 7.4) supplemented with 10% heat-inactivated FBS, 100 IU/mL of penicillin and 100 mg/mL of streptomycin (Palit & Ali 2008).

### Animal

BALB/c mice, 4–6 weeks old, weighing about 20 g, were procured from the National Centre for Laboratory Animal Sciences, Hyderabad, India. Animals were housed in an environmentally controlled room with 12 h light/dark cycle and fed standard diet and water *ad libitum* during experimental period. All animal experiments were carried out as per the approved guidelines of the Animal Ethics Committee of Tripura University (Registration Number: 1667/GO/a/12/CPSEA dated 12 November 2012), Suryamaninagar, Tripura, India.

### Determination of antileishmanial activity of methanol bark extracts of *S. villosa* against *L. donovani* promastigotes *in vitro*

The effect of SVE on the viability of *L. donovani* promastigotes was assessed by monitoring MTT [3-(4,5dimethylthiazol-2-yl)-2,5-diphenyltetrazolium bromide] assay. Stationary phase parasites were seeded at 2 × 10^6^ cells/mL cells per well in a 96-well flat bottom microtitre plate containing M199 medium. SVE with a graded concentrations starting from 0.5 to 130 μg/mL and reference standard drug AmpB with a IC_50_ value of 0.16 μg/mL (8 × 10^5^ cells/mL) (Dutta et al. 2007) were added and incubated at 22 °C for 72 h prior to the addition of MTT (20 μL per well of a 5 mg/mL MTT dye dissolved in 1% PBS solution) and then further incubated for 4 h. After incubation, 100 μL of SDS–HCl (10% SDS in 0.01 N HCl) in each well was added to dissolve the MTT formazan produced. The relative amount of formazan produced by viable cells per well was measured photometrically at 570 nm using a plate reader (Synergy H1 Hybrid Reader, Biotek, Winooski, VT) (Rudrapaul et al. 2014). The IC_50_ (concentration of compound needed for 50% inhibition of promastigote growth) value of SVE was determined from the graph representing different concentrations of the compounds plotted against % of promastigote cell viability.

### Analysis of antileishmanial activity by fluorescent microscopy

To visualize the effect of SVE on *L. donovani* promastigotes, 2 × 10^6^ cells/mL were taken in 35 × 10 mm tissue culture plate and incubated for 72 h with IC_50_ dose of SVE and IC_50_ dose of AmpB with respect to untreated promastigotes. After incubation, cell suspension was stained with 4 μg/mL dose of acridine orange for 15 min at dark. Following incubation, 10 μL of cell suspension was smeared over clean, dry glass slides and observed under fluorescent microscope (Leica DM 4000B, Wetzlar, Germany) using i3 filter. Images from 20 different fields were captured from smear of each treated cells.

### Measurement of reactive oxygen species levels

To monitor the level of ROS, SVE-treated and untreated *Leishmania donovani* AG83 promastigotes were used. Briefly, SVE-treated cells (10^7^ promastigotes/mL) were resuspended in 500 μL of M199 and labelled with H_2_DCFDA (2 μg/mL) for 15 min in the dark (Duranteau et al. 1998). Fluorimetric analyses at 507 nm excitation and 530 nm emission wavelengths were carried out in a Perkin Elmer spectrofluorometer (Waltham, MA). Data were obtained after subtraction of the basal fluorescence.

### Estimation of superoxide radical

To estimate the level of superoxide anion, 10^7^ promastigotes/mL was incubated with or without SVE and AmpB at IC_50_ doses for different time periods. The cells were then washed with PBS and resuspended in 100 μL PBS buffer. Each suspension (10 μL) was added to 1 mL of reaction mixture containing 50 mM sodium carbonate, 50 μM nitroblue tetrazolium (NBT), 0.1 mM EDTA and 0.5% Triton X-100 (Kono 1978). Analysis was carried out at 560 nm in a spectrophotometer (Shimadzu, Kyoto, Japan).

### Measurement of total fluorescent lipid peroxidation product

AG83 promastigotes (10^7^ cells/mL) treated and untreated with SVE and positive control AmpB at the IC_50_ doses for 1, 2, 3 and 4 h were pelleted down and washed twice with PBS. The pellet was suspended in 2 mL of 15% SDS in PBS solution. The fluorescence intensities of the total fluorescent lipid peroxidation products obtained from the SDS–promastigote interaction were estimated (Shimasaki 1994) with excitation at 360 nm and emission at 430 nm in a Perkin Elmer spectrofluorometer (Waltham, MA).

### DNA fragmentation assay

The assay was performed with minor modification as described previously (Sen et al. 2004; Dasgupta et al. 2008). Briefly, genomic DNA was isolated from the parasites (approximately 2.5 × 10^6^ cells/mL) after difierent treatments with or without SVE and AmpB at IC_50_ doses. The DNA was quantified and equivalent amount of DNA was electrophoresed in a 1% agarose gel at 75 V for 2 h and thereafter stained with EtBr and photographed under UV illumination.

### Isolation of peritoneal macrophages

Mouse macrophages were isolated by peritoneal lavage with ice-cold PBS, 48 h after intraperitoneal injection of 1.0 mL of sterile 4% thioglycolate broth. Cells were cultured as described previously (Bhattacharjee et al. 2009). The adherent cell population was cultured for 48 h prior to the study, to achieve the resting state.

### Cytotoxicity study

2 × 10^6^ cells/mL of peritoneal macrophages and murine macrophage cell line (RAW 264.7) were cultured in RPMI-1640 media supplemented with 10% FBS for 24 h in CO_2_ incubator at 37 °C. Following that, cells were incubated with IC_50_ doses of SVE and AmpB (as a reference drug) for 24 h at 37 °C. Thereafter, the medium was replaced with fresh RPMI (without Phenol Red) containing 1 mg/mL MTT. Cells were then incubated at 37 °C for 3 h, the untransformed MTT was removed and 50 μL of 0.04 M HCl–isopropanolic solution was added to each well. After 15 min of incubation at room temperature, absorbance was measured using an automatic plate reader (Synergy H1 Hybrid Reader, Biotek, Winooski, VT), at a reference wavelength of 690 nm and test wavelength of 650 nm (Mosmann 1983).

Murine blood (1 mL) was collected in heparin containing sterile tube and washed thoroughly (400×*g* for 10 min at 4 °C) with sterile phosphate-buffered saline (PBS: 6 mM KH_2_PO_4_; 30 mM Na_2_HPO_4_; 0.11 M NaCl; pH 7.4) to remove the buffy coat. The packed erythrocytes obtained at bottom were washed three times with sterile PBS and also suspended in PBS to obtain a cell suspension of 80 × 10^6^ cells/mL (Arizza et al. 2013). Aliquots of erythrocyte suspension (200 μL) were mixed with IC_50_ dose of SVE and IC_50_ dose of AmpB. After 1 h incubation at 37 °C, the reaction mixture was centrifuged at 800×*g* for 15 min at 4 °C to remove debris and residual erythrocytes. The OD of released haemoglobin was measured at 541 nm wavelength. Spontaneous haemoglobin release (0%) was estimated by incubating the RBCs with PBS while the complete haemolysis (100%) was assessed by incubating the RBCs in a solution of 0.1% Triton-X100 in distilled water. Haemolysis percentage was calculated according to the following equation:

Hemolysis(in%)={(ODofreleasedHbintheSVEtreatedsample)   -(ODofspontaneouslyreleasedHbintheuntreatedsample)/      ODofcompletelyreleasedHb}×100

### Toxicity study in BALB/c mice

For the toxicity study, five groups of animal were taken having five animals in each group. Animals were administered (intraperitoneal) with four different doses (25, 50, 100 and 200 mg/kg body weight) of SVE and a control group (DMSO) were included. The doses were administered at every one day interval for four consecutive weeks. Mice were then sacrificed and serum enzymes such as serum glutamate pyruvate transaminase (SGPT), serum glutamate oxaloacetate transaminase (SGOT), alkaline phosphatase (ALP), γ-glutamyl transferase (GGT), triglyceride (TG), cholesterol (CHL) and sugar (BS) were estimated. All serum parameters were analysed using kits from Dr. Reddy’s Laboratories (Hyderabad, India) following the manufacturer’s protocol.

### Particle size determination of methanol bark extract of *S. villosa* by atomic force microscopy (AFM)

Several concentrations of SVE viz. 10, 17.5 and 130 μg/mL were prepared in DMSO. Aliquot of each dilution (10 μL) was spread over clean dry glass coverslip as thin film and allowed to dry at 37 °C for 1 h. The films were observed under AFM (Bruker-Innova, Santa Barbara, CA) first at 20 μm scale and gradually up to 0.2 μm scale at a scanning speed of 1 Hz. All images were obtained with a resolution of 512 × 512 pixels. From the observed image, size distributions of particles were measured. All the experiments were performed in triplicate (Mukherjee et al. 2013).

### Statistical analysis

Each experiment was performed in triplicate on three different occasions. The values were the mean of three assays ± SD. Significance level was determined by using one way ANOVA and Student’s *t*-test. Data were presented as *p* value <0.01 (noted with *), *p* value <0.001 (noted with **) and *p* value <0.0001 (noted with ***). Statistical software Graph Pad Prism 6.0 (Graph Pad, San Diego, CA) was used for all statistical analysis.

## Results

### Phytochemical analysis of methanol bark extract of *S. villosa*

**Figure 1.**
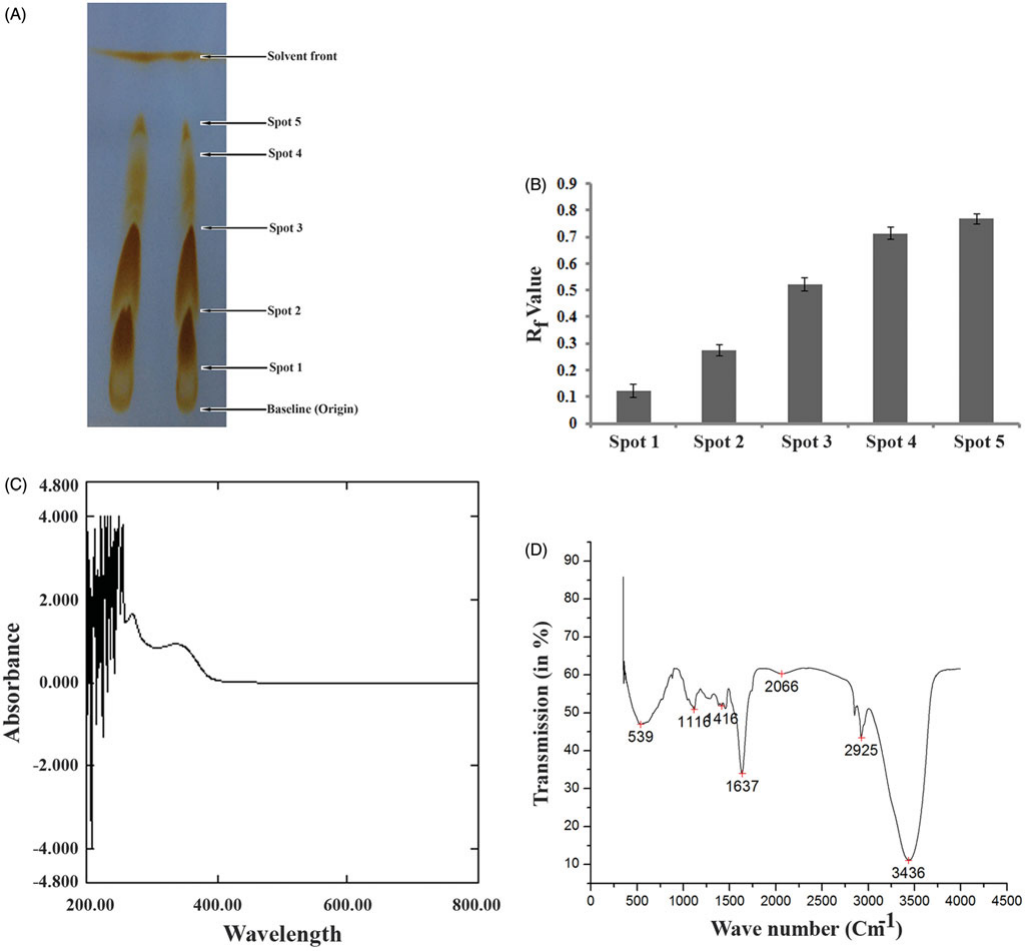
Estimation of presence of several phytoconstituents in SVE. (A) Thin layer chromatographic (TLC) separation of phytoconstituents in SVE. Presence of spot 1, 2, 3, 4 and 5 over TLC plate indicates presence of five different types of compounds in the extract. (B) Determined *R*_f_ value of each compound present in spot 1, 2, 3, 4 and 5 in TLC plate. *R*_f_ values were determined from the distance of spot 1, 2, 3, 4 and 5 with respect to the distance of solvent front of polar mobile phase from the origin point. Each value is the average of triplicate assay where presented data are mean ± SD. (C) UV–vis spectroscopic analysis of the SVE. Obtained graph shows several peaks in UV region which represents the presence of active phytochemicals in the extract. (D) IR spectroscopic analysis of SVE where obtained graph shows several peaks having different wave number. These represent several functional groups that are present in the SVE.

Presence of different types of phytochemicals in the SVE was determined by TLC, UV–Vis and IR spectroscopic analysis followed by several biochemical colour reactions. TLC of SVE produced five different distinct spots (Figure 1(A)) with *R*_f_ values ranging between 0.123 and 0.767 (Figure 1(B)). This signified that the spots on the TLC plate represented the individual phytochemicals present in SVE. To confirm the result of TLC, SVE was subjected to UV–Vis and IR spectroscopic study. Upon continuous measurement of absorbance from 200 to 800 nm, SVE showed several peaks in UV region (Figure 1(C)). This indicated that SVE contains several compounds that have absorption maximum in UV region. The IR spectrum showed the absorption peaks of hydroxyl (3436 cm^−1^), carbonyl (1637 cm^−1^), amines (1116 cm^−1^) and aromatic (1416 cm^−1^) functional groups (Figure 1(D)) in SVE. Presence of several functional groups signified that SVE contained several phyto-compounds. Taken together, the results of TLC and spectroscopic study inferred that SVE contains several phytochemicals. To assess the presence of different phytoconstituents in SVE, standard qualitative colour tests were performed. The positive results of the colour tests validated the presence of phytochemicals including flavonoids, alkaloids, tannin, sterol, anthraquinone, phenol and triterpene in methanol bark extract of *S. villosa* (Table 1).

**Table 1.** Qualitative phytochemical screening of SVE through several biochemical assays.

S. no.	Phytochemicals	Results
1	Flavonoids	**+**
2	Alkaloids	**+**
3	Tannin	**+**
4	Sterol	**+**
5	Anthraquinone	**+**
6	Phenol	**+**
7	Terpenoid	**+**
8	Anthocyanin	**_**
9	Reducing sugar	**_**
10	Quinone	**_**
11	Saponin	**_**

N.B. + indicates present; − indicates absent.

### Effect of methanol bark extract of *S. villosa* in the reduction of cell viability of *L. donovani* promastigotes

**Figure 2.**
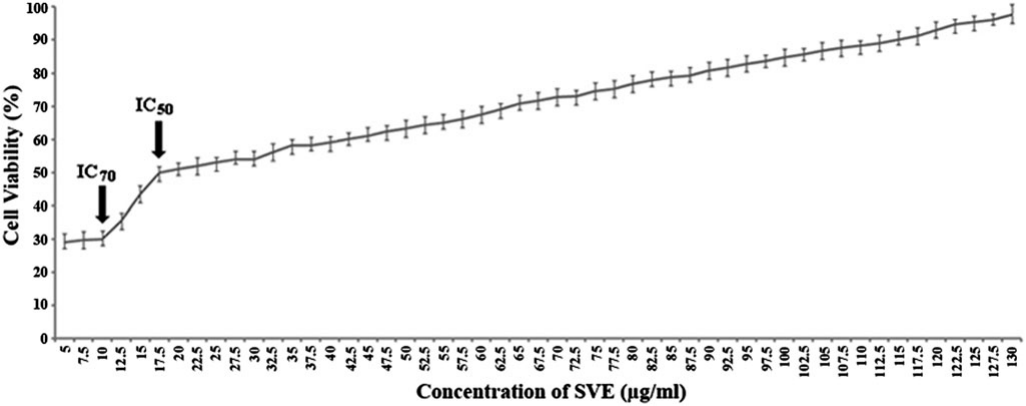
Antileishmanial activity of SVE against *Leishmania donovani* promastigotes were determined by MTT assay. Viable cell numbers were plotted against concentration of SVE and IC_50_ dose of 17.5 μg/mL was determined where maximum activity of SVE occurs at 10 μg/mL (IC_70_). Each value is the average of triplicate assay where presented data are mean ± SD.

Leishmanicidal activity of the SVE was studied with doses ranging from 0 to 130 μg/mL on the promastigotes of *L. donovani* parasites. The viability of *L. donovani* promastigotes was determined by MTT assay (Figure 2). The conversion of tetrazolium salt to an insoluble formazan product by the mitochondrial electron transport chain is an indicator of promastigote viability while a decrease in the amount of formazan indicates toxicity to the *Leishmania* promastigotes. The concentration of SVE that inhibited 50% growth of the stationary phase promastigotes was found to be 17.5 μg/mL (IC_50_). Whereas, SVE at a dose of 10 μg/mL showed maximum inhibitory activity where it killed almost 70% promastigotes. Exposure of the promastigotes to SVE at doses less than 10 μg/mL up to 5 μg/mL showed almost similar pattern of killing. Further reduction of doses (<5 μg/mL) abruptly reduced the killing ability of SVE. The cell viability pattern was found to be similar to untreated promastigotes. Though doses of 130–0 μg/mL were tested, the most potent reverse dose dependent antileishmanial activity of SVE is demonstrated at IC_50_ and IC_70_ values of 17.5 and 10 μg/mL, respectively (Figure 2).

**Figure 3.**
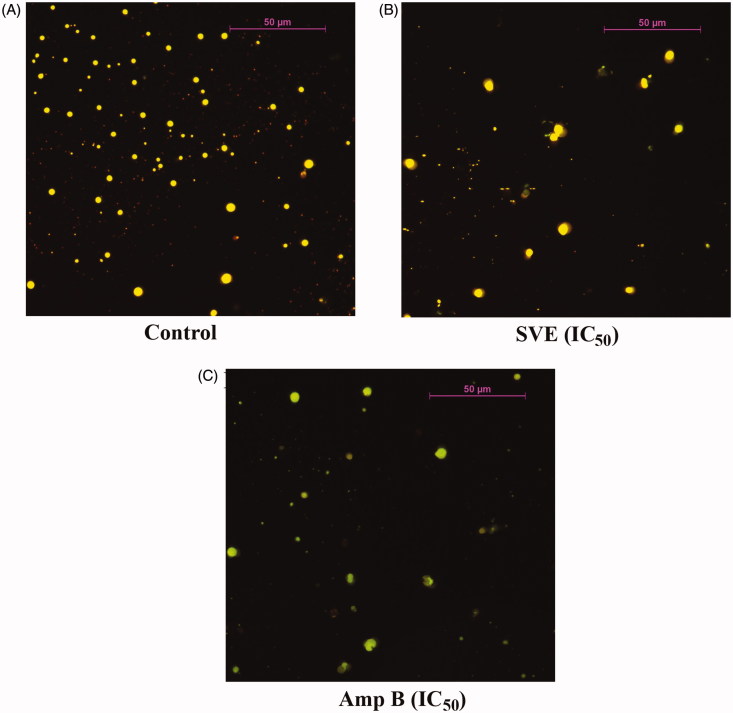
Antileishmanial activity of SVE against *Leishmania donovani* promastigotes was visualized through observation under fluorescence microscope after staining with acridine orange. Result shows that *Leishmania donovani* promastigote numbers were significantly reduced after treatment with IC_50_ dose of SVE (B), and IC_50_ dose of standard drug amphotericin B (C) with respect to untreated control (A). Pictures were obtained from 20 different fields of the same treatment and best pictures were presented here.

To further validate the antileishmanial activity of SVE, *L. donovani* promastigotes were treated with SVE and observed under fluorescence microscope after staining with acridine orange. Under fluorescence microscope, antileishmanial activity of SVE (at IC_50_ dose) with respect to standard antileishmanial agent AmpB (IC_50_ dose) was studied (Figure 3(C)). Interestingly observation under fluorescent microscope showed that at IC_50_ concentration (Figure 3(B)) of SVE, there was significant inhibition of *Leishmania* parasites with respect to untreated control (Figure 3(A)). This validated the *in vitro* leishmanicidal activity of SVE.

### Induction of lipid peroxidation during oxidative stress

**Figure 4.**
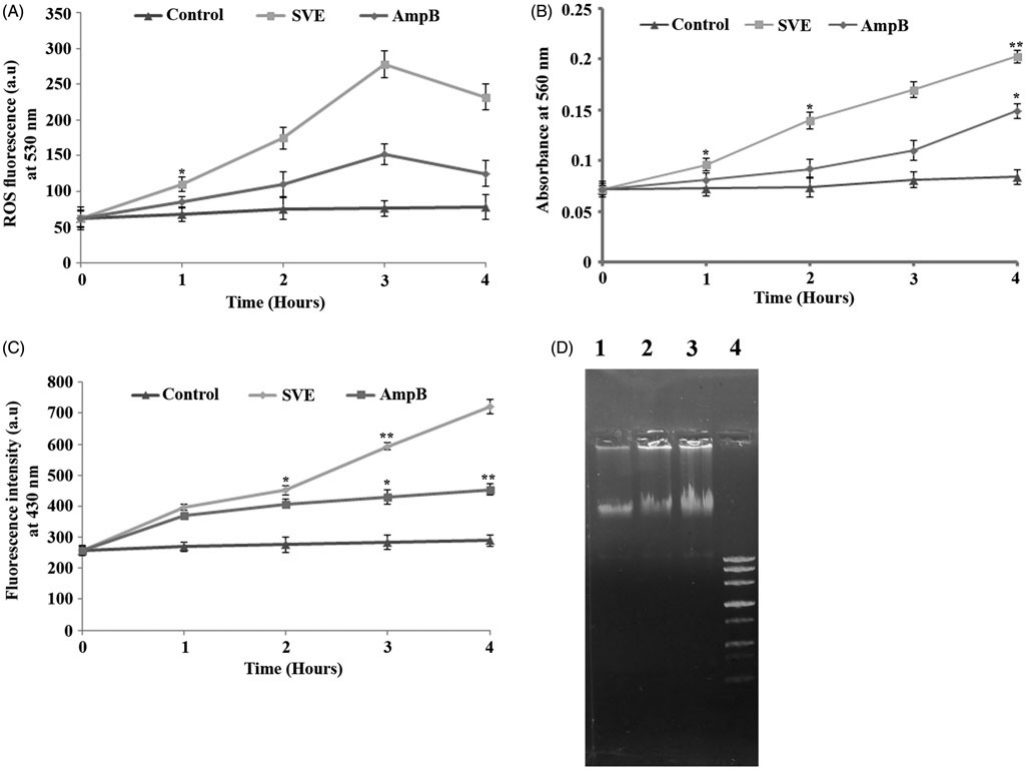
Determination of ROS, superoxides, lipid peroxidation level and genomic instability of promastigotes. Measurement of ROS production after treatment of AG83 promastigotes with IC_50_ dose of SVE (A). Determination of SVE (IC_50_ dose) treated superoxide generation after treatment of AG83 promastigotes (B). The level of fluorescent products of lipid peroxidation was measured after treatment of AG83 promastigotes with IC_50_ dose of SVE (C). Each value is the average of triplicate assay where presented data are mean ± SD. Statistical analysis was done using one way ANOVA, *p value <0.01 and **p value <0.001 (*n* = 3). Fragmentation of genomic DNA in the presence of IC_50_ dose of SVE compared to untreated control. Genomic DNA was isolated from AG83 promastigotes after 2 h treatment of SVE (lane 2), IC_50_ dose of standard drug AmpB (lane 3) compared with untreated control (lane 1) and DNA ladder (lane 4) (D).

*L. donovani* promastigotes were treated with the IC_50_ dose of SVE to investigate the generation of ROS. The increase in the fluorescence intensity of the treated cells in comparison to untreated controls signified physiological production of ROS by the mitochondrion. It was observed that the IC_50_ dose of SVE induces ROS generation up to 3 h, whereas after 3 h of treatment, the levels of ROS significantly declined with increasing time (Figure 4(A)). ROS induced oxidative burst is an early event which was also seen after SVE treatment and reached a peak value at about 3 h. Considering that oxidative burst occurs for a very short time (Das et al. 2001), it can be inferred that ROS may trigger SVE-induced cell death (Fonseca-Silva et al. 2011).

Cellular stress induced by SVE led to the formation of intracellular superoxide radicals, as evident by the reduction of NBT to blue formazan in the promastigote cell lysates. It was found out that superoxide radical level in SVE-treated cells remained higher than the untreated control throughout the study (Figure 4(B)). Further, lipid peroxidation was quantified by measuring the total fluorescent lipid peroxide products in promastigotes after treatment with SVE at different time points. Observed results demonstrated upregulation of lipid peroxidation at 1 h post treatment, reached a maximum level at 4 h (Figure 4(C)). Hence, it is evident that the ROS generation caused by SVE-induces oxidative stress which in turn influenced the increased level of lipid peroxidation.

### Methanol bark extracts of *S. villosa* induced DNA fragmentation in *L. donovani* promastigotes

Agarose gel electrophoresis of DNA isolated from SVE treated cells showed significant fragmentation of DNA (Figure 4(D), lane 2) in comparison to cells treated with IC_50_ dose of AmpB (Figure 4(D), lane 3) and untreated control (Figure 4(D), lane 1).

### Detection of nanosized particle by AFM

**Figure 5.**
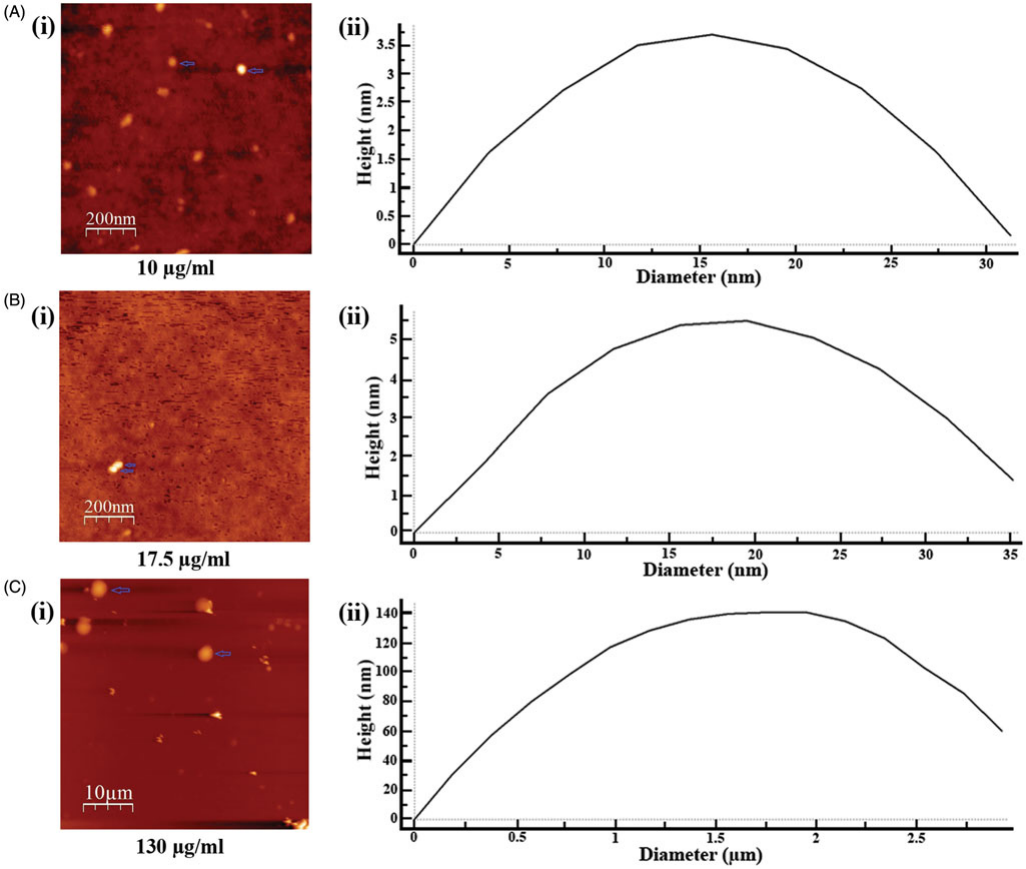
Determination of particle size of phytoconstituents present in SVE by atomic force microscope. Gradient concentrations of SVE were spread as film, observed under atomic force microscope and presented the image of observed particle with particle size distribution graph. Image and particle size of compounds at 10 μg/mL (A(i) and (ii)), 17.5 μg/mL (B(i) and (ii)) and 130 μg/mL (C(i) and (ii)) explain that with increase in concentration of SVE particle size of compounds increases.

To explore the underlying cause of reverse dose dependent antileishmanial activity of SVE, the size distribution of molecular aggregates present in 10, 17.5 and 130 μg/mL concentrations of SVE was studied by AFM. It was observed that 130 μg/mL concentration contained an average size of 2.93 μm in diameter which is quite big for transport within cell (Figure 5(C)(i)(ii)). For 17.5 μg/mL concentration, the average compound aggregate size was 35.2 nm in diameter, which was smaller than the previous set (Figure 5(B)(i)(ii)). In 10 μg/mL concentration, average particle size was 31.3 nm (Figure 5(A)(i)(ii)). This indicated that in 10 μg/mL concentration, aggregated compounds were smallest in size but with increase in concentration, size of aggregates increased accordingly.

### Cytotoxicity study of methanol bark extract of *S. villosa*

**Figure 6.**
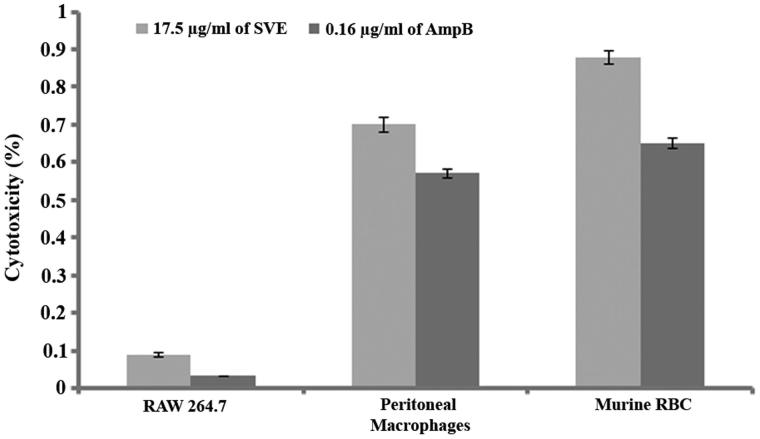
Percentage cytotoxicity of 17.5 μg/mL of SVE and 0.16 μg/mL of amphotericin B against RAW 264.7 macrophages, murine peritoneal macrophages and murine RBC was determined. Each value is the average of triplicate assay where presented data are mean ± SD.

The above mentioned bioactivity study revealed that SVE possesses potent antileishmanial activity. While targeting these doses for therapeutic application on human host, all these doses should execute very low cytotoxicity. In this regard, the level of cytotoxicity was assessed in RAW 264.7 cell line, murine peritoneal macrophages and murine RBCs by IC_50_ dose of SVE with reference to IC_50_ dose of Amp-B. It was observed that IC_50_ dose of SVE executed 0.09% and 0.7% cytotoxicity against RAW 264.7 cell line and murine peritoneal macrophages, respectively (Figure 6). Whereas, AmpB at IC_50_ dose executed 0.032% and 0.57% toxicity against RAW 264.7 cell line and murine peritoneal macrophages, respectively (Figure 6). It was also found that SVE at IC_50_ dose executed 0.88% cytotoxicity whereas Amp-B at IC_50_ dose executes 0.65% cytotoxicity on murine RBCs (Figure 6).

In addition, to further assess the *in vivo* toxicity of SVE, liver function enzymes such as SGOT, SGPT, ALP, GGT and other associated parameters including TG, CHL and BS were quantified. It was observed that in mice treated up to 100 mg/kg body weight dose of SVE, the serum liver function enzyme profile and other parameters (TG, CHL and BS) showed similar pattern to that of untreated control (Table 2). Interestingly, along with other parameters, the blood sugar level was seen to gradually increase with respect to control in doses above 100 mg/kg body weight. All these data together justify that, up to a dose of 100 mg/kg body weight, SVE is nontoxic to mouse liver. As a whole, it can be inferred that SVE at its IC_50_ dose is nontoxic to murine RBCs, murine peritoneal macrophage and macrophage cell line RAW 264.7 and up to a dose of 100 mg/kg body weight SVE does not cause any *in vivo* toxicity to mouse liver.

**Table 2.** Analysis of liver function and other serum parameters from mouse serum by biochemical colour test.

Concentration of SVE(mg/kg body weightof mouse)	Quantity of liver function and other serum parameters in mouse serum						
	SGOT (IU/L)	SGPT (IU/L)	ALP (IU/L)	GGT (IU/L)	TG (mg/dL)	CHL (mg/dL)	BS (mg/dL)
0	14.342 ± 1.052	7.072 ± 0.879	10.852 ± 1.09	4.632 ± 0.082	135.48 ± 9.34	39.88 ± 1.96	138.58 ± 7.89
25	13.842 ± 1.271	6.827 ± 0.996	10.603 ± 0.998	4.142 ± 0.121^a^	133.89 ± 7.76	37.14 ± 3.73	137.20 ± 3.72
50	13.996 ± 1.063	6.942 ± 1.221	10.699 ± 2.321	4.232 ± 0.982	134.26 ± 5.91	37.73 ± 2.84	137.56 ± 2.93
100	14.179 ± 1.233	7.136 ± 1.094	10.782 ± 1.281	4.615 ± 0.994	135.29 ± 4.87	38.97 ± 1.93^a^	138.05 ± 4.56
200	16.216 ± 1.337	9.009 ± 1.937	28.933 ± 2.993^b^	7.174 ± 0.163	148.89 ± 4.96^a^	47.55 ± 1.99	159.59 ± 5.43^a^

N.B.: SVE: *Sterculia villosa* methanolic bark extract; SGPT: serum glutamate pyruvate transaminase; SGOT: serum glutamate oxaloacetate transaminase; ALP: alkaline phosphatase; GGT: γ-glutamyl transferase; TG: triglyceride; CHL: cholesterol; BS: blood sugar. All data are presented as value ± SD. Statistical analysis was done using ANOVA and Student’s *t*-test.

a *p* value <0.01.

b *p* value <0.001 (*n* = 3) with respect to untreated control.

## Discussion

Visceral leishmaniasis, also known as kala-azar is a parasitic disease caused by *L. donovani*. Pathogenicity of this particular disease is associated with fever, cachexia, hepatosplenomegaly, blood cytopenia and immune suppression (Carvalho et al. 1981, 1985). Recently several trials are on-going throughout the globe to design a potent antileishmanial agent with less toxicity. At present, though there are some antileishmanial drugs in clinical use like sodium stibogluconate, meglumine antimoniate, AmpB and miltefosine, etc. but surprisingly, the parasite gradually becomes resistant to these compounds (Chappuis et al. 2007). Therefore, search of new potent antileishmanial agent is the urgent need to protect the mankind from leishmaniasis. Plant extracts or plant-derived compounds are likely to provide a valuable source for new therapeutic agents (Kayser & Kiderlen 2001; de Carvalho & Ferreira 2007). The urgent need of alternative strategies has led to the screening of natural products for their potential use in the treatment of leishmaniasis. The leishmanicidal activity from methanol extracts of several plants has been evaluated (Rocha et al. 2005). In the present study, SVE was prepared to assess the presence of phytoconstituents and its antileishmanial activity. Previously it has been reported that *Sterculia villosa* possessed ethnomedicinal properties against skin diseases (Kunwar et al. 2010), inflammation (Hossain et al. 2012), microbial infection (Tania et al. 2013), etc. In this study for the first time, it has been reported that the methanol bark extract of *S. villosa* possesses antileishmanial potential against promastigotes form of *L. donovani*. In this particular study, the antileishmanial potential of SVE was evaluated against *L. donovani* promastigotes in comparison with standard antileishmanial drug amphotericin-B.

Plants produce several secondary metabolites as metabolic end products which mostly get deposited in the stem bark of the plant (Godghate & Sawant 2014). Therefore, the bark was selected for the present study. In this direction, SVE was analysed for the presence of different types of phytochemicals by TLC, UV–Visible, IR spectroscopic study and confirmed by biochemical colour tests before studying its bioactivity. For identification of different phytoconstituents, at first, SVE was subjected to thin layer chromatography over silica gel surface. After developing the spot on silica gel, SVE gave five different distinct bands (Figure 1(A)) with different *R*_f_ values (Figure 1(B)). Literature suggests that different compounds differ in their *R*_f_ values based on their partition distribution co-efficient, therefore it can be assumed that SVE does contain several phytoconstituents. This was further validated by UV–Vis and IR spectroscopic study followed by biochemical colour tests. In case of UV–Vis absorption from 200 to 800 nm, it was found that there were several peaks in UV region (below 400 nm) which can be attributed to the presence of UV active phytochemicals (Figure 1(C)). Plant pigments generally give absorption peaks in visible region, so the peaks below the visible range validate the presence of active phytoconstituents (Mlodzinska 2009; Neha & Jyoti 2013). In connection with presence of phytochemicals, the SVE was further examined for the presence of functional groups through IR spectroscopy and it was found that the extract contained hydroxyl (3436 cm^−1^), primary amine/aliphatic, amines/nitrogenous (1637 and 1116 cm^−1^) and aromatic (1416 cm^−1^) functional groups (Figure 1(D)). Presence of several functional groups indicated that SVE certainly contains various phytochemicals. To, further justify these findings; several biochemical colour reactions were also performed. In these tests, specific phytochemicals like alkaloids, glycosides, flavonoids, etc. formed specific colour complexes or products that produce a coloured solution in the test tube (De et al. 2010; Firadouse & Alam 2011; Ganatra et al. 2012; Yusuf et al. 2014). These colour reactions are qualitatively specific for respective phytoconstituents (Table 1). Based on these findings, the antileishmanial activity of SVE against *L. donovani* promastigotes was confirmed.

The antileishmanial activity of SVE was compared with standard antileishmanial drug, AmpB against extracellular promastigote form of *L. donovani*. SVE showed inhibitory effect against promastigote form of *L. donovani* when doses of 130–0 μg/mL were tested (Figure 2), the most potent reverse dose dependent antileishmanial activity was demonstrated at IC_50_ and IC_70_ values of 17.5 and 10 μg/mL, respectively. IC_50_ value of SVE with respect to AmpB was further validated by observation of promastigotes under fluorescent microscope after staining with acridine orange (Figure 3). In this case all live promastigotes after SVE treatment appeared as green in colour. From this observation, it can be inferred that SVE has reverse dose dependent antileishmanial potential against promastigote form of parasite. This indicated that SVE executed higher activity at lower concentrations. Wherein, below the 5 μg/mL concentration, SVE does not show any significant activity which is almost similar to the untreated cells. This might be due to the fact that the threshold concentration of the constituent compounds required for bioactivity is present in the extract at 5 μg/mL concentration. Whereas doses less than 5 μg/mL concentration may not contain optimum quantity of active compounds in SVE, as a result does not execute any bioactivity (Lagunin et al. 2000; Sarethy et al. 2015).

The IC_50_ value of SVE against promastigotes causes an increase in cellular ROS production where there is a gradual increase up to 3 h which contains a peak value, after which the ROS production declines. Persistence of viable promastigotes shows a gradual decline with the increase of ROS (Das et al. 2001). Further, superoxide generation and lipid peroxidation levels also get elevated with time where the peak values were observed in 4 h (Figure 4). The DNA fragmentation of promastigotes occurred after 2 h of SVE treatment which supports the induction of cell death (Gamcsik et al. 2001) (Figure 4). To explore the underlying cause of reverse dose dependent antileishmanial activity, the size distribution of molecular aggregation present in 10, 17.5 and 130 μg/mL concentrations was also found by AFM. It was observed that particle sizes were smallest at 10 μg/mL and comparatively bigger at 130 μg/mL of SVE (Figure 5). This is a unique property of nanosize compounds to form bigger aggregates at higher concentration. The reason behind the formation of these nanosized particles with dilution is not known to us and needs further study on size reduction. Formation of bigger size aggregates may reduce leishmanial membrane permeability which in turn may reduce their bioactivity as well (Morones et al. 2005). Thus, SVE at 10–17.5 μg/mL concentration exhibits higher leishmaniacidal activity in comparison to higher concentrations. The mechanism of particle size reduction of methanol plant extract with dilution is not reported yet. But execution of higher bioactivity due to smallest nanosized particle at lower concentration of compounds has already been reported in literature (Morones et al. 2005; Harrison et al. 2008; Simitsopoulou et al. 2014). It was also reported that the presence of many molecules together may mask the activity of active compounds (Harrison et al. 2008). Thus, higher dilution can reduce concentration of interfering substances which in turn increase the bioactivity of the phytoconstituents. In the present investigation, size distribution study reveals that with dilution up to 10 μg/mL concentration, the constituent particles were in their smallest size whereas with the increase in the concentration of SVE to 17.5 μg/mL, the particle size of the constituents also increased. Likewise, due to the presence of smallest size particles in 10 μg/mL concentration, SVE exhibited increased antileishmanial activity (IC_70_) in comparison with 17.5 μg/mL (IC_50_ dose).

The experimental data indicated that SVE has stronger inhibitory effect against promastigotes form of the parasite. Based on these findings SVE can be projected for therapeutic application. To project the SVE for therapeutic application SVE should have permitted level of toxicity. In this direction, the level of cytotoxicity was evaluated with IC_50_ dose of SVE on murine macrophage cell line (RAW 264.7), murine peritoneal macrophages and murine RBCs. It was found that the IC_50_ dose of SVE and IC_50_ dose of AmpB executed very less toxicity (<0.9%) which is very much within the permitted limit (Figure 6). Extent of toxicity was further validated by estimation of liver function panel of enzymes, CHL, TG, etc. from murine serum. As liver is the prime and foremost organ involved in metabolism, thus any change in liver tissue may lead to pathophysiologic manifestation in body. Thus to determine any such change in the liver function, enzyme profile was quantified. All these enzymes (i.e., SGOT, SGPT, ALP and GGT) are physiological markers of liver function. In addition to assess the extent of lipid and protein metabolism by liver, the level of TG and CHL was quantified from serum. To further validate the physiology or pathological change of liver tissue, the serum sugar content was also quantified, as utilization of sugar by any cell directly relates to the physiology of that cell. Once cell undergoes any pathophysiologic change, this will lead to reduced utilization of sugar. As a result, serum sugar content rises with time. In the present study, it was found out that the level of serum parameters rises after dose of 100 mg/kg body weight (Table 2). This signifies that SVE up to 100 mg/kg body weight dose is nontoxic to mouse.

## Conclusions

Observed results of the present study suggest that the SVE contains different phytochemicals. SVE has executed reverse dose dependent, potent antileishmanial activity against *L. donovani* promastigotes with IC_50_ value at 17.5 μg/mL. SVE treated promastigotes also showed elevated level of cellular ROS production, increased superoxide generation, upregulation of lipid peroxidation and DNA fragmentation, which indicate the parasitic cell death. It was found that increasing size of molecular aggregation with rise in concentration is the reason behind reverse dose dependent activity. Through detailed *in vitro* and *in vivo* toxicity study we also can infer that SVE is not toxic to mouse liver (up to 100 mg/kg body weight dose) and against peritoneal macrophages as well as RAW 264.7 macrophage cell line (at IC_50_ dose). So in summary, it can be concluded that *Sterculia villosa* methanol bark extract may be further explored as a therapeutic agent against VL.
